# *Nectria*-related fungi causing dieback and canker diseases in China, with *Neothyronectriacitri* sp. nov. described

**DOI:** 10.3897/mycokeys.56.36079

**Published:** 2019-07-10

**Authors:** Qin Yang, Wen-Yan Chen, Ning Jiang, Cheng-Ming Tian

**Affiliations:** 1 The Key Laboratory for Silviculture and Conservation of the Ministry of Education, Beijing Forestry University, Beijing 100083, P.R. China Beijing Forestry University Beijing China

**Keywords:** DNA phylogeny, *
Nectriaceae
*, Systematic, Taxonomy

## Abstract

To clarify phylogenetic relationships amongst *Nectria*, *Neothyronectria* and *Thyronectria* in *Nectriaceae*, we examined detailed morphological characters and performed phylogenetic analyses of a concatenated dataset, based on the ITS, LSU, *tef1* and *tub2* DNA sequences of fungal specimens in China. Four species of nectria-related fungi were identified, i.e. *Nectriadematiosa*, *N.pseudotrichia*, *Neothyronectriacitri* and *Thyronectriapinicola*. The newly described species, *Neothyronectriacitri*, is characterised by its ascomatal wall with bright yellow scurf, unitunicate asci, each with 4-spored and ascospores allantoid to short-cylindrical, uniseriate, muriform, hyaline to slightly yellowish-brown. This species has affinities with other one known species of *Neothyronectria* and can be distinguished by molecular data.

## Introduction

*Nectriaceae* Tul. & C. Tul., typified by the genus *Nectria* (Fr.) Fr., was established by Tulasne and Tulasne (1865) to include nectria-related fungi having brightly pigmented ascomata with fusiform to allantoid ascospores and globose to fusiform phialidic conidia (Rossman et al. 1999, 2013, Rossman 2000, Lombard et al. 2015, Maharachchikumbura et al. 2015, Huang et al. 2018, Yang et al. 2018). Members of the family are unified by phenotypic characters such as uniloculate ascomata that are yellow, orange-red to purple and phialidic asexual morphs. Lombard et al. (2015) defined the generic concepts in *Nectriaceae*, based on a multi-gene phylogenetic analysis and resolved 47 genera supported by morphological observations. Since then, *Neothyronectria* was proposed as a new genus to accommodate the species, *Neothyronectriasophorae*, which is known only from the pycnidial asexual morph (Crous et al. 2016) and *Cosmosporella* was proposed as a new genus (Huang et al. 2018), thus 49 genera are now accepted in the *Nectriaceae*.

*Nectria*, typified by *N.cinnabarina* (Tode: Fr.) Fr., was initially established by Fries (1849). Some species of *Nectria* are weak parasites of woody plants (Samuels et al. 2009, Hirooka et al. 2011). Hirooka et al. (2012) reviewed the genus, based on the type and additional herbarium specimens, and accepted 29 species. They also monographed the genus *Thyronectria* as *Pleonectria* but because *Thyronectria* (1875) is older, it has priority over *Pleonectria* (1876) as explained by Jaklitsch and Voglmayr (2014). Many members of *Nectria* and *Thyronectria* occur on dead corticated twigs or branches of woody plants worldwide mainly in temperate and subtropical regions (Hirooka et al. 2012, Jaklitsch and Voglmayr 2014, Zeng and Zhuang 2016). To date, 42 species of *Thyronectria* have been accepted (Jaklitsch and Voglmayr 2014, Voglmayr et al. 2016, Zeng and Zhuang 2016, Lechat et al. 2018).

During trips to collect forest pathogens in China, several nectria-related fungi associated with canker or dieback diseases were collected. Based on a multi-locus phylogeny (ITS, LSU, *tef1* and *tub2*), we identified four nectria-related species in three genera of *Nectriaceae* and propose one new species in *Neothyronectria*.

## Materials and methods

### Isolates

Fresh specimens were collected from infected branches or twigs of diverse hosts from Beijing, Heilongjiang, Jiangxi, Shaanxi and Xinjiang provinces, China. Strains were isolated from fresh diseased branches and grown from ascospores or conidia by spreading the suspension on the surface of 1.8% potato dextrose agar (PDA), incubated at 25 °C for up to 24 h. Single germinating conidia were removed and transferred to fresh potato dextrose agar (PDA) plate. Specimens and isolates of the new species have been deposited in the Museum of Beijing Forestry University (BJFC). Axenic cultures are maintained in the China Forestry Culture Collection Center (CFCC).

### Morphological analysis

Morphological observations of the sexual and asexual morph in the natural environment were based on features of the fruiting bodies produced on infected plant tissues and micromorphology, supplemented by cultural characteristics. Gross morphology of fruiting bodies was recorded using a Leica stereomicroscope (M205 FA). Perithecia, pycnidia, synnemata and stromata were observed and described. To test ascomatal wall reactions, 3% KOH and 100% lactic acid (LA) were used. The micromorphological characteristics were examined by mounting fungal structures in clear lactic acid and 30 measurements at 1000× magnification were determined for each isolate using a Leica compound microscope (DM 2500) with differential interference contrast (DIC) optics. Colony characters and pigment production on PDA were noted after 10 d. Colony colours were described according to Rayner (1970). Longitudinal descriptions, nomenclature and illustrations of taxonomic novelties are deposited in MycoBank (http://www.MycoBank.org; Crous et al. 2004).

### DNA extraction, PCR amplification and sequencing

Genomic DNA was extracted from colonies grown on cellophane-covered PDA, using a modified CTAB [cetyltrimethylammonium bromide] method (Doyle and Doyle 1990, Zhang et al. 2010). For PCR amplifications of phylogenetic markers, four different primer pairs were used (Table 1). PCR amplification products were assayed via electrophoresis in 2% agarose gels. DNA sequencing was performed using an ABI PRISM 3730XL DNA Analyzer with a BigDye Terminater Kit v.3.1 (Invitrogen, USA) at the Shanghai Invitrogen Biological Technology Company Limited (Beijing, China).

**Table 1.** Genes used in this study with PCR primers, process and references.

**Gene**	**PCR primers (forward/reverse)**	**PCR: thermal cycles: (Annealing temp. in bold)**	**References of primers used**
ITS	ITS1/ITS4	(95 °C: 30 s, **51 °C**: 30 s, 72 °C: 1 min) × 35 cycles	White et al. 1990
LSU	LROR/ LR5	(95 °C: 45 s, **55 °C**: 45 s, 72 °C: 1 min) × 35 cycles	Vilgalys and Hester 1990, Rehner and Samuels 1994
*tef1*	EF1-728F and EF-1567R	(95 °C: 15 s, **55 °C**: 20 s, 72 °C: 1 min) × 35 cycles	Carbone and Kohn 1999, Rehner 2001
*tub2*	T1/T2	(95 °C: 30 s, **55 °C**: 30 s, 72 °C: 1 min) × 35 cycles	O’Donnell and Cigelnik 1997

### Phylogenetic analyses

The quality of our amplified nucleotide sequences was checked and combined by SeqMan v.7.1.0 and reference sequences were retrieved from the National Center for Biotechnology Information (NCBI), according to recent publications of the family *Nectriaceae* (Jaklitsch and Voglmayr 2014, Lombard et al. 2015, Crous et al. 2016, Yang et al. 2018). Sequences were aligned using MAFFT v. 7.310 (http://mafft.cbrc.jp/alignment/server/index.html) (Katoh and Standley 2016) and manually corrected using Bioedit 7.0.9.0 (Hall 1999).

Phylogenetic analyses of the combined gene regions were performed using Maximum Parsimony (MP), Maximum-Likelihood (ML) and Bayesian Inference (BI) methods. The data were edited in AliView version: 1.19-beta1k and the evolutionary model obtained using MrModeltest v. 2.3 (Nylander et al. 2008) under the Akaike Information Criterion (AIC) performed in PAUP v. 4.0b10. The MP analysis was performed by a heuristic search option of 1000 random-addition sequences with a tree bisection and reconnection (TBR) algorithm. Maxtrees were set to 5000, branches of zero length were collapsed and all equally parsimonious trees were saved. Other calculated parsimony scores were tree length (TL), consistency index (CI), retention index (RI) and rescaled consistency (RC). ML was performed using RAxML-HPC v.8 on XSEDE in CIPRES Science Gateway (Miller et al. 2010, 2015, Stamatakis 2014) with 1000 rapid bootstrap replicates using the GTR+I+G model of nucleotide substitution. BI was implemented by MrBayes v. 3.0b4 (Ronquist and Huelsenbeck 2003) with GTR+I+G as the best-fit model. Posterior Probabilities (PP) were estimated by Markov Chain Monte Carlo sampling (MCMC) in MrBayes v. 3.0b4 (Huelsenbeck and Ronquist 2001). Two MCMC chains, started from random trees for 1,000,000 generations and trees, were sampled every 100^th^ generation, resulting in a total of 10,000 trees. The first 25% of trees were discarded as the burn-in phase of each analysis. Branches with significant Bayesian Posterior Probabilities (BPP) were estimated in the remaining 7500 trees. Phylogenetic trees were viewed with FigTree v.1.3.1 (Rambaut and Drummond 2010) and processed by Adobe Illustrator CS5. Alignment and trees were deposited in TreeBASE (submission ID: 24366). The nucleotide sequence data of the new taxon have been deposited in GenBank (Table 1).

## Results

### Phylogenetic analyses

To reveal the phylogenetic position amongst *Nectria*, *Neothyronectria* and *Thyronectria* in *Nectriaceae*, a phylogenetic analysis was performed with combined ITS, LSU, *tef1* and *tub2* sequence data. Sequences of representative species were selected from NCBI (Jaklitsch and Voglmayr 2014, Crous et al. 2016, Yang et al. 2018). The ITS, LSU, *tef1*, *tub2* and combined data matrices contained 545, 781, 1033, 643 and 3010 characters with gaps, respectively. The alignment comprised 59 strains and *Emericellopsisglabra* (CBS 125295), *Hydropisphaerafungicola* (CSB 122304), *Nectriopsisexigua* (CBS 126110) and *Verrucostomafreycinetiae* (MAFF 240100) were selected as the outgroups.

**Table 2.** Strains and GenBank accession numbers of the isolates used in this study.

Species	Isolate No.	Substrate/Host	Country	GenBank Accession No.			
				ITS	LSU	*tef1*	*tub2*
* Allantonectria miltina *	CBS 121121	* Agave americana *	Italy	HM484547	HM484572	HM484524	HM484609
* Emericellopsis glabra *	CBS 125295	Soil	Mexico	HM484860	GQ505993	HM484843	HM484879
* Hydropisphaera fungicola *	CBS 122304	Decaying leaves on *Populustrichocarpa*	USA	HM484863	GQ505995	HM484845	HM484877
* N. antarctica *	CBS 115033	* Berberis aquifolium *	USA	HM484556	HM484560	HM484516	HM484601
* N. asiatica *	MAFF 241439	Bark of dead wood	Japan	HM484701	HM484563	–	HM484604
* N. aurantiaca *	CBS 308.34	*Ulmus* sp.	UK	JF832628	JF832682	JF832519	JF832886
* N. balansae *	CBS 123351	*Coronilla* sp.	France	HM484552	GQ505996	HM484525	HM484607
* N. balansae *	CBS 129349	Twigs	China	JF832653	JF832711	JF832522	JF832908
* N. berberidicola *	CBS 128669	* Berberis vulgaris *	France	JF832662	JF832712	JF832538	JF832887
* N. cinnabarina *	CBS 125165	Dead twigs of *Aesculus* sp.	France	HM484548	HM484562	HM484527	HM484606
*N.dematiosa* Subclade A	CBS 126570	Bark	USA	HM484557	HM484561	HM484534	HM484603
***N.dematiosa* Subclade A**	**CFCC 53585**	*** Tilia mandshurica ***	**China**	**MK861084**	**MK861075**	**MK902792**	**MK902801**
***N.dematiosa* Subclade A**	**CFCC 53586**	*** Betula platyphylla ***	**China**	**MK861085**	**MK861076**	**MK902793**	**MK902802**
*N.dematiosa* Subclade B	CBS 125125	Dead twigs of *Acermacrophyllum*	Canada	HM484676	HM484717	HM484645	HM484797
* N. eustromatica *	CBS 121896	–	–	HM534896	HM534896	HM534875	–
* N. eustromatica *	CBS 125578	–	–	HM534897	HM534897	HM534876	–
* N. magnispora *	CBS 129362	–	Japan	JF832663	JF832683	JF832539	JF832896
* N. magnispora *	CBS 129361	Twigs	Japan	JF832664	JF832685	JF832540	JF832897
* N. mariae *	CBS 125294	* Buxus sempervirens *	France	JF832629	JF832684	JF832542	JF832899
* N. nigrescens *	CBS 125148	Dead twigs of dicotyledonous tree	USA	HM484707	HM484720	HM484672	HM484806
* N. nigrescens *	CBS 128988	* Elaeagnus angustifolia *	USA	JF832630	JF832687	–	JF832888
* N. nigrescens *	CBS 129808	* Ulmus pumila *	USA	JF832632	JF832690	–	JF832894
* N. polythalama *	CBS 128672	Twigs	New Zealand	JF832638	JF832695	JF832523	JF832900
* N. pseudocinnabarina *	CBS 129366	Dead wood	Venezuela	JF832642	JF832697	JF832533	–
* N. pseudotrichia *	CBS 551.84	Bark	Japan	HM484554	GQ506000	HM484532	HM484602
* N. pseudotrichia *	MAFF 241452	Bark	Japan	JF832649	JF832706	JF832531	JF832903
* N. pseudotrichia *	G.J.S. 09-1329	Dead wood	Venezuela	JF832647	JF832702	JF832530	JF832902
*** N. pseudotrichia ***	**CFCC 53587**	***Robinia* sp**.	**China**	**MK861086**	**MK861077**	**MK902794**	**MK902803**
*** N. pseudotrichia ***	**CFCC 53588**	*** Cinnamomum porrectum ***	**China**	**MK861087**	**MK861078**	**MK902795**	**MK902804**
*** N. pseudotrichia ***	**CFCC 53589**	*** Rubus corchorifolius ***	**China**	**MK861088**	**MK861079**	**MK902796**	**MK902805**
* N. sordida *	CBS 125119	Living woody vine	French Guiana	HM484857	HM484868	HM484848	HM484874
* N. triseptata *	HAMS 252485	On rotten twig	China	KM026503	KM026504	KM026506	KM026501
* N. ulmicola *	CFCC 52117	Ulmus davidiana var. japonica	China	MG231959	MG231980	MG232022	MG232043
* N. ulmicola *	CFCC 52118	Ulmus davidiana var. japonica	China	MG231960	MG231981	MG232023	MG232044
* Nectriopsis exigua *	CBS 126110	* Myxomycete *	Puerto Rico	HM484865	GQ506014	HM484852	HM484883
*** Neothyronectria citri ***	**CFCC 53590**	***Citrusmaxima* cv. *Shatian***	**China**	**MK861080**	**MK861071**	**MK902788**	**MK902797**
*** N. citri ***	**CFCC 53591**	***Citrusmaxima* cv. *Shatian***	**China**	**MK861081**	**MK861072**	**MK902788**	**MK902798**
* N. sophorae *	CBS 142094	* Sophora microphylla *	Zew Zealand	KY173470	KY173559	–	KY173619
* Thyronectria aquifolii *	CBS 307.34	* Ilex aquifolium *	UK	JF832597	JF832718	JF832548	JF832842

The concatenated sequence alignment contained 932 parsimony-informative characters, 259 were variable and parsimony uninformative and 1819 were constant. The parsimony analysis yielded the maximum of 10 equally most parsimonious trees (TL = 5493 steps; CI = 0.386; RI = 0.685; RC = 0.264; HI = 0.614).

**Figure 1.**
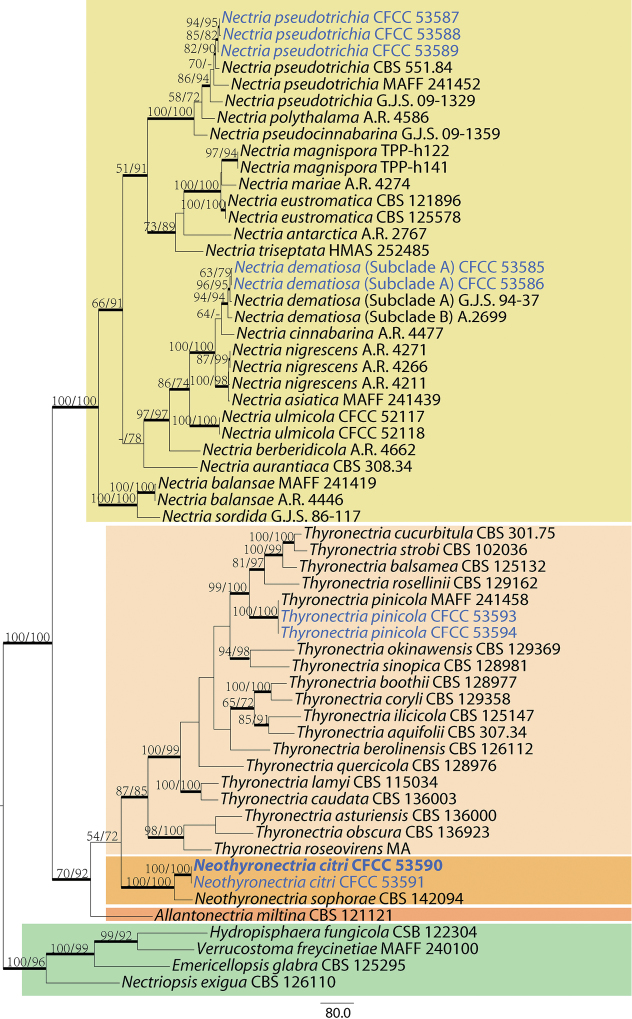
Maximum parsimony phylogenetic tree generated from analysis of a combined ITS, LSU, *tef1* and *tub2* sequence dataset for 59 taxa of *Allantonectria*, *Nectria*, *Neothyronectria* and *Thyronectria*. *Emericellopsisglabra* (CBS 125295), *Hydropisphaerafungicola* (CSB 122304), *Nectriopsisexigua* (CBS 126110) and *Verrucostomafreycinetiae* (MAFF 240100) as outgroup taxa. Values above the branches indicate maximum parsimony and maximum likelihood bootstrap (left, MP BP ≥ 50%; right, ML BP ≥ 50%). The branches with significant BIPP values (≥ 0.95) in the BI analysis are thickened. Scale bar = 80 nucleotide substitutions. Strains in current study are in blue. Ex-type strains are indicated in bold.

The phylogeny, resulting from the MP analysis of combined gene sequence data, is shown in Fig. 1. Overall, the topologies obtained from the different phylogenetic analyses were mostly similar and the best scoring MP tree is illustrated here. The MP and ML bootstrap support values above 50% are shown at the first and second position, respectively. Branches with significant BPP (≥ 0.95) in Bayesian analyses were thickened in the phylogenetic tree.

### Taxonomy

#### Nectria

Taxon classificationFungiHypocrealesNectriaceae

(Fr.) Fr., Summa veg. Scand., Sectio Post. (Stockholm): 387, 1849

##### Type species.

*Nectriacinnabarina* (Tode) Fr., Summa veg. Scand., Sectio Post. (Stockholm): 388, 1849.

##### Note.

Members of *Nectria* are typically weak parasites of woody plants and occur on hardwood trees and shrubs throughout the temperate zone of the northern hemisphere (Samuels et al. 2009, Hirooka et al. 2011). The genus *Nectria* is characterised by well-developed stromata, subglobose to globose, red to dark red, fleshy, soft-textured, uniloculate, warted perithecia that become cupulate when dry and are associated with coelomycetous asexual morphs. Asci are unitunicate and clavate to cylindrical in shape. Ascospores are variable and usually broadly ellipsoid to long-fusiform, hyaline to yellow brown, smooth to striate and non- to multi-septate or muriform (Rossman et al. 1999, Hirooka et al. 2009, Maharachchikumbura et al. 2015).

#### Nectria dematiosa

Taxon classificationFungiHypocrealesNectriaceae

(Schwein.) Berk., Grevillea 4: 16, 1875

Fig. 2

##### Description.

See Yang et al. (2018)

##### Additional specimens examined.

CHINA. Heilongjiang Province, Liangshui Nature Reserve, 47°10'50.64"N, 128°53'41.03"E, on twigs or branches of *Tiliamandshurica* Rmpr.et Maxim., 29 July 2016, Q. Yang (BJFC-S1400, living culture CFCC 53585); Xinjiang, 45°13'07.97"N, 81°46'24.71"E, on twigs or branches of *Betulaplatyphylla* Suk., 18 July 2017, C.M. Tian (BJFC-S1767, living culture CFCC 53586).

##### Note.

*Nectriadematiosa* has a broad host range and is widely distributed in China, occurring as the most commonly *Nectria* species (Yang et al. 2018). This study is the first report of *N.dematiosa* from *Betulaplatyphylla* and *Tiliamandshurica*.

**Figure 2.**
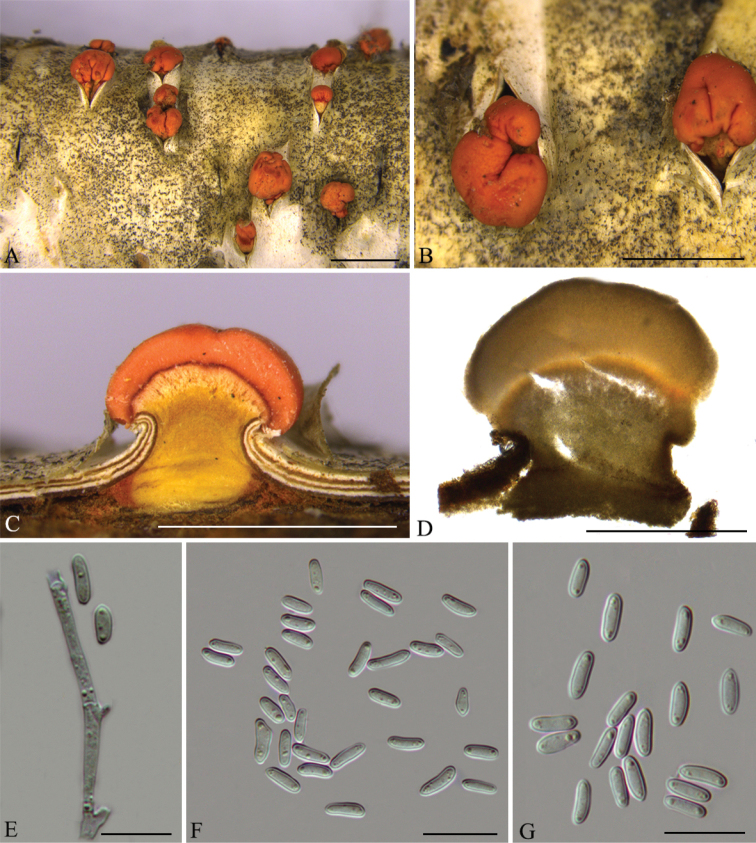
*Nectriadematiosa* (CFCC 53585) **A–B** habit of conidiomata on branches **C** transverse section of conidioma **D** longitudinal section of conidioma **E** conidiophores **F–G** conidia. Scale bars: 1 mm (**A–C**); 500 μm (**D**); 10 μm (**E–G**).

#### Nectria pseudotrichia

Taxon classificationFungiHypocrealesNectriaceae

Berk. & M.A. Curtis, J. Acad. Nat. Sci. Philadelphia 2, 2: 289. 1853

Fig. 3

##### Description.

See Yang et al. (2018)

##### Additional specimens examined.

CHINA. Shaanxi Province, Ankang City, 32°40'32.85"N, 109°18'57.38"E, on twigs or branches of *Robinia* sp., 29 July 2016, N. Jiang (BJFC-S1403, living culture CFCC 53587); Jiangxi Province, Ganzhou City, 24°40'51.80"N, 115°31'49.99"E, on twigs or branches of *Cinnamomumporrectum* (Roxb.) Kosterm., 12 May 2018, Q. Yang (BJFC-S1768, living culture CFCC 53588); Jiangxi Province, Ganzhou City, 24°59'44.81"N, 115°30'58.85"E, on twigs or branches of *Rubuscorchorifolius* Linn. f., 12 May 2018, Q. Yang (BJFC-S1769, living culture CFCC 53589).

##### Note.

*Nectriapseudotrichia* is one of the common tropical fungi in the genus *Nectria* and is distinguished in the genus by having muriform ascospores and a synnematous asexual morph.

**Figure 3.**
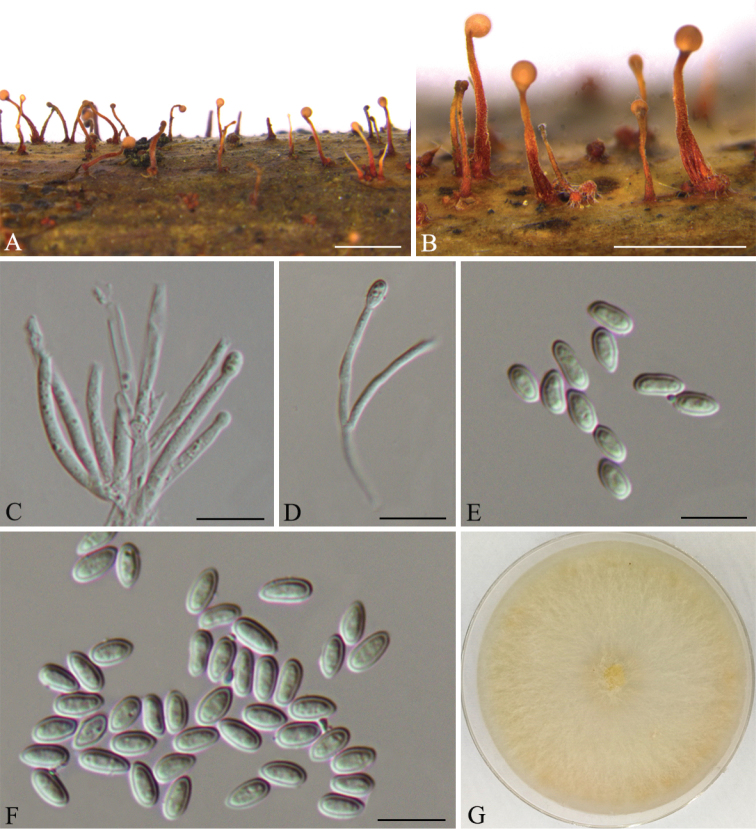
*Nectriapseudotrichia* (CFCC 53587) **A–B** habit of conidiomata on branches **C–D** conidiophores **E–F** conidia. Scale bars: 1 mm (**A–B**); 10 μm (**C–F**).

#### Neothyronectria

Taxon classificationFungiHypocrealesNectriaceae

Crous & Thangavel, Persoonia 37: 329, 2016.

##### Type species.

*Neothyronectriasophorae* Crous & Thangavel, Persoonia 37: 329, 2016.

##### Note.

The genus *Neothyronectria* was described by Crous & Thangavel (2016) based on the only species, *N.sophorae*, which is known from a pycnidial asexual morph. *Neothyronectria* is characterised by pycnidial conidiomata that exude a creamy mucoid conidial mass and hyaline, ampulliform to subcylindrical conidia. In this study, we collected and illustrated here one additional taxon in *Neothyronectria*.

#### Neothyronectria citri

Taxon classificationFungiHypocrealesNectriaceae

C.M. Tian & Q. Yang sp. nov.

830779

Figure 4

##### Diagnosis.

*Neothyronectriacitri* differs from its closest phylogenetic neighbour *Neothyronectriasophorae* in ITS, LSU and *tub2* loci, based on the alignments deposited in TreeBASE.

##### Holotype.

CHINA. Jiangxi Province: Ganzhou city, 25°51'27.87"N, 114°58'18.95"E, on symptomatic branches of *Citrusmaxima* (Burm.) Merr. cv. *Shatian* Yu, 11 May 2018, Q. Yang, Y.M. Liang & Y. Liu (holotype BJFC-S1770 designated here, ex-type culture CFCC 53590).

##### Etymology.

Named after the host genus on which it was collected, *Citrus*.

##### Description.

*Mycelium* not visible around ascomata or on the host. *Stromata* erumpent through epidermis, up to 0.6 mm high and 1 mm diam., pseudoparenchymatous, cells forming *textura angularis* to *t. globulosa*, intergrading with ascomatal wall. *Ascomata* superficial on well-developed stromata, scattered to aggregated in groups of 3–10, subglobose to globose, 200–270 μm diam., rarely slightly cupulate upon drying, sometimes with only a depressed apical region, yellowish-brown to grey, apical region slightly darker, no colour change in KOH or LA, sometimes surface scurfy or scaly, bright yellow to greenish-yellow. *Ascomatal surface cells* forming *textura globulosa* or *t. angularis*, sometimes including bright yellow scurf, 9–15 μm diam., walls pigmented, uniformly about 1.5 μm thick. *Ascomatal wall* 27–46 μm thick, of two regions: outer region 22–35 μm thick, intergrading with stroma, cells forming *textura globulosa* or *t. angularis*, walls pigmented, about 1.5 μm thick; inner region 9–15 μm thick, of elongate, thin-walled, hyaline cells, forming *textura prismatica*. *Asci* clavate, unitunicate, 53.5–65 × 8.5–11 μm, with inconspicuous ring at apex, 4-spored. *Ascospores* allantoid to short-cylindrical, uniseriate, rounded at both ends, (17–)18–21(–23.5) × 8–9(–10) μm (n = 20), muriform, hyaline to slightly yellowish-brown.

##### Culture characters.

Cultures incubated on PDA at 25 °C in darkness. Colony originally flat with white aerial mycelium, becoming pale yellowish due to pigment formation, conidiomata absent.

##### Additional specimen examined.

CHINA. Jiangxi Province: Ganzhou City, 25°51'27.87"N, 114°58'18.95"E, on symptomatic branches of *Citrusmaxima* (Burm.) Merr. cv. *Shatian* Yu, 11 May 2018, Q. Yang, Y.M. Liang & Y. Liu (BJFC-S1771, living culture CFCC 53591).

##### Note.

*Neothyronectriacitri*, as described here, is known from an ascomatal sexual morph phylogenetically allied to species of *Allantonectria* and *Thyronectria* (Fig. 1). In this study, two strains representing *Neothyronectriacitri* cluster in a well-supported clade and appear most closely related to *Neothyronectriasophorae*, which was isolated from *Sophoramicrophylla* in New Zealand (Crous et al. 2016). *Neothyronectriacitri* can be distinguished, based on ITS, LSU and *tub2* loci from *Neothyronectriasophorae* (16/464 in ITS, 9/772 in LSU and 60/494 in *tub2*).

**Figure 4.**
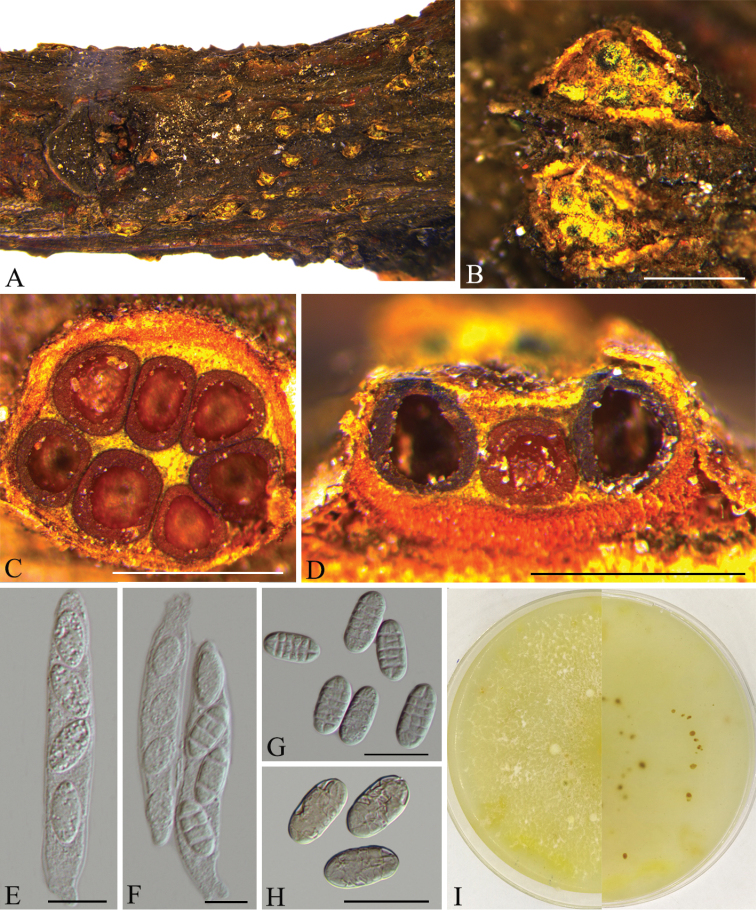
*Neothyronectriacitri* (CFCC 53590) **A–B** habit of conidiomata on branches **C** transverse section of conidioma **D** longitudinal section of conidioma **E–F** asci **G–H** ascospores. Scale bars: 500 μm (**B–D**); 10 μm (**E–H**).

#### Thyronectria

Taxon classificationFungiHypocrealesNectriaceae

Sacc., Grevillea 4: 21, 1875.

##### Type species.

*Thyronectriarhodochlora* (Mont.) Seeler, J. Arnold Arbor. 21: 455, 1940.

##### Note.

*Thyronectria* Sacc. was established by Saccardo (1875) to include nectria-like fungi with immersed ascomata and muriform ascospores and characterised by well-developed erumpent stromata which are often covered with yellow-green amorphous scurf and ascospores that sometimes bud in the ascus to produce ascoconidia (Jaklitsch and Voglmayr 2014, Lombard et al. 2015). Members of the genus occur on dead corticated twigs or branches of woody plants worldwide mainly in temperate and subtropical regions (Hirooka et al. 2012, Jaklitsch and Voglmayr 2014).

#### Thyronectria pinicola

Taxon classificationFungiHypocrealesNectriaceae

(Kirschst.) Jaklitsch & Voglmayr, Persoonia 33: 203, 2014.

Figure 5

##### Basionym.

*Pleonectriapinicola* Kirschst., Abh. Bot. Ver. Prov. Brandenburg 48: 59, 1906.

##### Description.

*Stromata* erumpent through epidermis, orange to red. *Pycnidia* solitary or aggregated in groups of 3–6, superficial on stroma or rarely immersed at base, subglobose, smooth to slightly roughened, cerebriformis or slightly cupulate upon drying, 225–400 μm high, 240–440 μm diam., red to bay, KOH+ slightly darker, LA+ slightly yellow. *Pycnidial wall* 16–40 μm thick, of two regions: outer region 11–15 μm thick, intergrading with stroma, cells forming *textura globulosa* or *t. angularis*, walls pigmented, about 1.5 μm thick; inner region 10–24 μm thick, of elongate, thin-walled, hyaline cells, forming *textura prismatica*. *Conidiophores* densely branched, generally with 1–3 branches, 8.5–24 μm long, 1.3–1.5 μm wide. *Conidiogenous cells* cylindrical monophialides on aerial, submerged or repent hyphae. *Conidia* formed abundantly on slimy heads, ellipsoidal to oblong, hyaline, straight, rounded at both ends, non-septate, (2–)3–3.5 × 0.7–1.0 μm (n = 20), smooth-walled.

##### Culture characters.

Cultures incubated on PDA at 25 °C in darkness. Colony surface cottony with aerial mycelium, becoming yellowish-brown due to pigment formation, small reddish-brown sporodochial conidial masses produced after 3–4 wk.

##### Specimens examined.

CHINA. Beijing: Chaoyang District, 40°00'35.31"N, 116°47'55.32"E, on symptomatic branches of PinussylvestrisLinn.var.mongolica Litv., 11 June 2018, Q. Yang & N. Jiang (BJFC-S1773, living culture CFCC 53593 and CFCC 53594).

##### Note.

The hosts of *Thyronectriapinicola*, synonymised with *Pleonectriapinicola*, are restricted to *Pinus*. Members of the genus distributed in Asia (China, Japan, Pakistan), Australia, Europe (Germany, Russia), North America (USA) and South America (Chile) (Jaklitsch and Voglmayr 2014). The asexual morph of *T.pinicola* in the natural environment has long, sterile hyphae extending from the hymenium and abundant conidiophores (Figs 4E–G). In the present study, two isolates from twigs of Pinussylvestrisvar.mongolica were congruent with *T.pinicola*, based on morphology and DNA sequences data (Fig. 1). We therefore describe *T.pinicola* as a known species for this clade.

**Figure 5.**
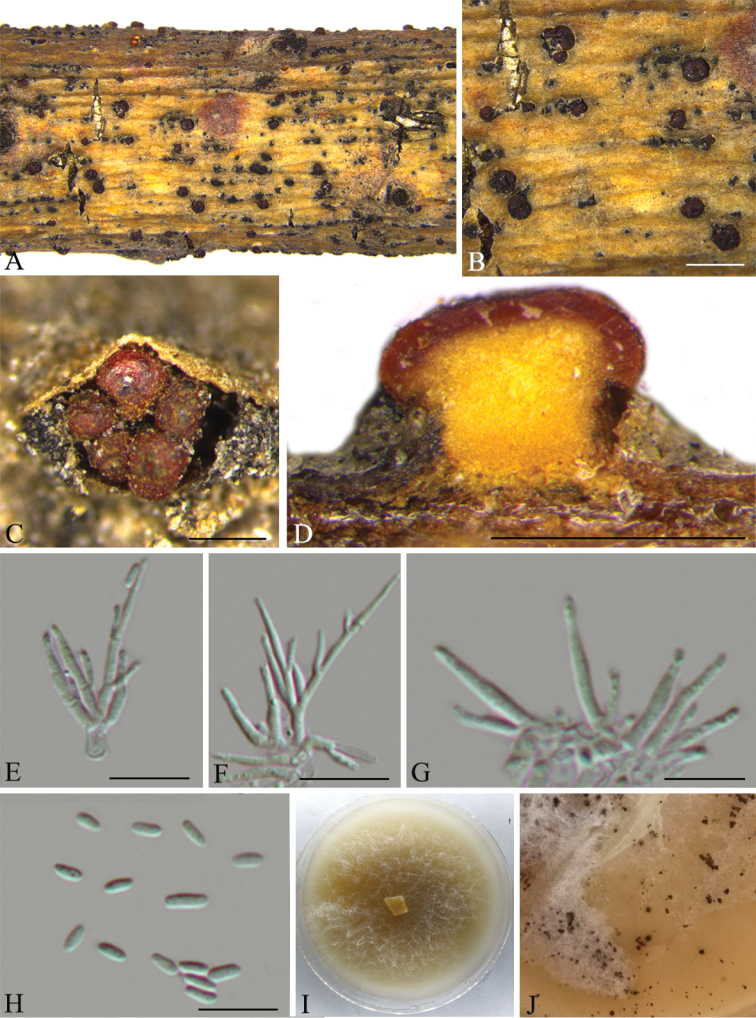
*Thyronectriapinicola* (CFCC 53593) **A–C** habit of conidiomata on branches **D** longitudinal section of conidioma **E–G** conidiogenous cells with conidia **H** conidia **I–J** culture on PDA and conidiomata. Scale bars: 1 mm (**B**); 500 μm (**C–D**); 10 μm (**E–H**).

## Discussion

In this investigation of nectria-related fungi in China, we identified four species in three genera (*Nectria*, *Neothyronectria* and *Thyronectria*) of *Nectriaceae*, based on four combined loci (ITS, LSU, *tef1* and *tub2*), as well as morphological characters. It includes *Nectriadematiosa*, *N.pseudotrichia*, and *Thyronectriapinicola* as well as one new species named *Neothyronectriacitri*. The new species is characterised by well-developed erumpent stromata that are often covered with yellow-green amorphous scurf; asci unitunicate, clavate, with inconspicuous ring at apex, each with 4-spored; ascospores allantoid to short-cylindrical, uniseriate, muriform, hyaline to slightly yellowish.

Species revised by Rossman et al. (1999) in *Nectria* were monographed by Hirooka et al. (2012), who recognised three genera, i.e. *Allantonectria*, *Nectria* and *Pleonectria*. *Allantonectria*, based on *Allantonectriamiltina*, was recognised as a monotypic genus with small, aseptate ascospores, trichoderma-like conidiophores and occurring on monocotyledonous plants. The genus *Thyronectria* (as *Pleonectria*) is characterised by having ascomata with bright yellow scurf, ascospores that often bud to produce ascoconidia inside or outside of the asci and/or a pycnidial anamorph (Hirooka et al. 2012). Based on the lack of bright yellowish scurf on the ascomata, the genus *Nectria* is easily distinguished from *Allantonectria* and *Thyronectria*. In this study, *Neothyronectriacitri* was identified as a new species in *Neothyronectria*, which was typified by *Neothyronectriasophorae* having ampulliform to subcylindrical conidia (Crous et al. 2016). Unlike species of *Thyronectria*, *Neothyronectria* did not produce ascoconidia but they have bright yellow scurf on the ascomatal wall.

In the taxonomy of hypocrealean fungi, the reaction of the perithecial wall to KOH is considered as an important character (Rossman et al. 1999, Zeng and Zhuang 2016). Most species of *Allantonectria* and *Thyronectria* have perithecial colour turning darker to blood-red or purple in KOH. However, some species in *Thyronectria* display a weak or negative reaction to KOH, which might be influenced by the presence of scurf covering the perithecia or their dark-coloured ascomata (Hirooka et al. 2012, Jaklitsch and Voglmayr 2014, Zeng and Zhuang 2016). In our study, the dark perithecial walls of *Neothyronectriacitri* do not change colour in KOH but the major features, such well-developed stromata and ascomata with bright yellow scurf, as well as the molecular data, also provide strong evidence that it belongs to *Neothyronectria*.

## Supplementary Material

XML Treatment for Nectria

XML Treatment for Nectria dematiosa

XML Treatment for Nectria pseudotrichia

XML Treatment for Neothyronectria

XML Treatment for Neothyronectria citri

XML Treatment for Thyronectria

XML Treatment for Thyronectria pinicola
